# Baricitinib in the Treatment of Severe Atopic Dermatitis Resistant to Cyclosporine: A Case Report Involving Two Cases

**DOI:** 10.7759/cureus.79638

**Published:** 2025-02-25

**Authors:** Abhishek De, SK Shahriar Ahmed, Disha Chakraborty, Azhar Khan, Sandipan Dhar

**Affiliations:** 1 Dermatology, Calcutta National Medical College and Hospital, Kolkata, IND; 2 Dermatology, Institute of Child Health, Kolkata, IND

**Keywords:** atopic dermatitis, atopic eczema, baricitinib, cyclosporin, jak inhibitor

## Abstract

Atopic dermatitis (AD) is a chronic inflammatory skin disorder that profoundly affects quality of life, particularly in patients with moderate-to-severe disease that is refractory to conventional treatments. Cyclosporine is a cornerstone of systemic therapy for severe AD; however, its long-term use is hindered by toxicity, and effective alternatives are often unavailable or insufficient. Janus kinase inhibitors, including baricitinib, have emerged as promising therapeutic options by modulating key inflammatory pathways involved in AD pathogenesis. This report describes two cases of severe cyclosporine-refractory AD that were successfully managed with baricitinib. Both patients showed substantial clinical improvement, including significant reductions in affected body surface area, disease severity scores, pruritus, and sleep disturbances within four weeks of initiating treatment. No serious adverse effects were observed, and the treatment was well tolerated. These findings highlight the potential of baricitinib as a viable therapeutic alternative for severe AD, particularly in settings where biologics such as dupilumab or abrocitinib may be limited. While baricitinib demonstrates both rapid and sustained efficacy, further studies are warranted to confirm its long-term safety and define its optimal role in AD management.

## Introduction

Atopic dermatitis (AD) is a prevalent inflammatory skin disorder affecting up to 3% of adults and 20% of children in high-income nations. Moderate-to-severe cases constitute 15%-50% of occurrences, contributing to a substantial economic burden and a marked decline in quality of life [1]. Indian guidelines recommend cyclosporine at 3-5 mg/kg for severe AD. However, safe and effective alternative options remain limited when cyclosporine fails or is contraindicated [2,3].

Emerging targeted molecular therapies are revolutionizing dermatological treatment. The Janus kinase-signal transducer and activator of transcription (JAK-STAT) pathway is a crucial signaling cascade where various proinflammatory pathways converge. Many inflammatory skin conditions are driven by soluble inflammatory mediators that depend on the JAK-STAT signaling pathway. Therefore, blocking this pathway with JAK inhibitors (JAKi) could be beneficial for such diseases [4]. Currently, seven JAKi are used in the treatment of AD, namely abrocitinib, baricitinib, gusacitinib, upadacitinib, tofacitinib, delgocitinib, and ruxolitinib. However, as of 2022, only abrocitinib and upadacitinib have received FDA approval for the treatment of AD. Among these, baricitinib became the first JAKi to receive European approval for AD in 2020 [5,6]. Despite being available for rheumatologic conditions in India, baricitinib has not yet been approved for AD. Here, we present two male patients with severe cyclosporine-refractory AD who achieved symptom control with baricitinib after multiple conventional therapies proved ineffective.

## Case presentation

Case 1

A 16-year-old boy presented with a chronic history of moderate-to-severe AD since early childhood. His condition showed minimal response despite multiple treatments, including high-potency topical and systemic corticosteroids, narrowband ultraviolet B phototherapy, cyclosporine, and tofacitinib.

At presentation, about 73% of his body was affected, manifesting as thickened, excoriated plaques with marked lichenification and severe xerosis. His quality of life was significantly impaired, as reflected in a high Dermatology Life Quality Index (DLQI) score of 17 and a Scoring AD (SCORAD) severity score of 64. Sleep disturbances due to intense pruritus were significant, with the patient rating both itching and insomnia as 6 out of 10.

**Figure 1:**
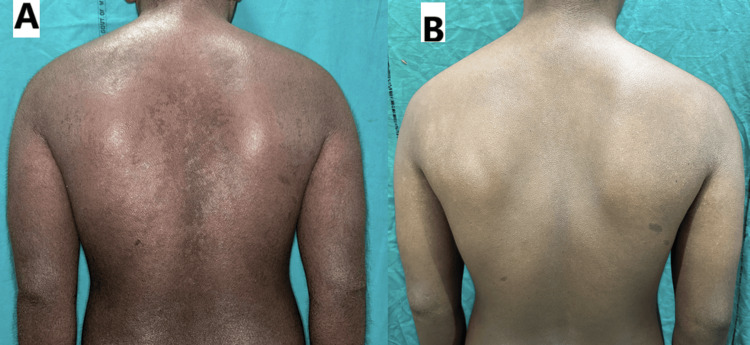
The clinical images of the first patient (A) before and (B) after treatment with baricitinib showing distinct improvement in erythema and lichenification

A comprehensive laboratory evaluation yielded normal results, including complete blood count, erythrocyte sedimentation rate, C-reactive protein, and renal and liver function tests, while tuberculosis screening was negative. After obtaining informed consent, treatment was initiated with baricitinib (2 mg twice daily), alongside levocetirizine (5 mg nightly) and twice-daily emollient application. Follow-up assessments were scheduled on days 14, 28, and 42 to monitor clinical response and adverse effects. The patient showed significant improvement over 42 days, with affected body surface area (BSA) decreasing from 73% on day 1 to 16% on day 42 (Figure 1).

Key clinical indicators, including DLQI (from 17 to 4), SCORAD (from 64 to 8), Eczema Area and Severity Index (EASI; from 32 to 6), Severity of Pruritus Scale (SPS; from 3 to 1), sleep disturbance (from 6 to 2), and numerical itch scale (from 7 to 2), all demonstrated marked reductions, reflecting substantial symptomatic relief (Table 1).

**Table 1:** The follow-up data pertaining to BSA encompassing various scoring systems for AD concerning the first patient during baricitinib therapy BSA: body surface area; DLQI: Dermatology Life Quality Index; SCORAD: Scoring Atopic Dermatitis; EASI: Eczema Area and Severity Index; SPS: Severity of Pruritus Scale; AD: atopic dermatitis

Follow-up	BSA involvement	DLQI	SCORAD	EASI	SPS	Sleep disturbance	Numerical itch scale
Day 1	73%	17	64	32	3	6	7
Day 14	73%	14	48	25	2	5	5
Day 28	43%	8	21	11	1	3	3
Day 42	16%	4	8	6	1	2	2

Case 2

A 42-year-old man presented with a chronic, debilitating case of adult-onset AD that had persisted for 10 years since the age of 32. Despite multiple treatments, including topical and systemic corticosteroids, phototherapy, and immunosuppressants such as cyclosporine and tofacitinib, he experienced only transient relief, with symptoms rebounding upon treatment tapering. At the time of consultation, he was receiving cyclosporine (250 mg daily) but continued to show signs of disease progression.

On examination, AD covered 65% of his body, characterized by thickened, excoriated plaques, severe lichenification, and pronounced xerosis. His condition significantly impaired his quality of life, reflected in a DLQI score of 20 and a SCORAD of 55. He reported severe pruritus and sleep disturbances, rating both at 7 out of 10.

**Figure 2:**
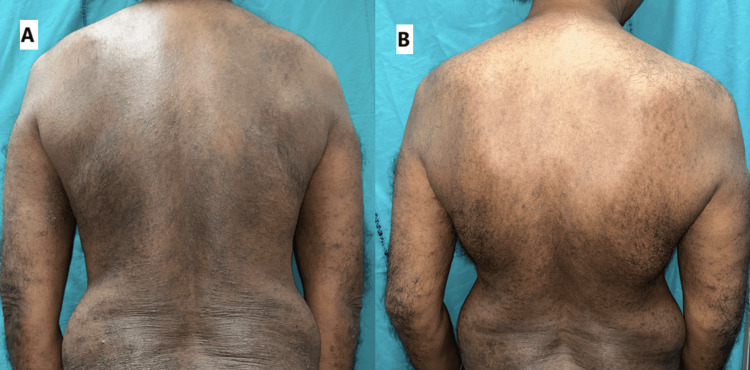
The clinical images of the second patient (A) before and (B) after treatment with baricitinib depicting improvement in AD signs AD: atopic dermatitis

Routine laboratory investigations and tuberculosis screening yielded normal results. Following informed consent, treatment with baricitinib (2 mg twice daily), cetirizine (10 mg once daily), and regular emollient application was initiated. Follow-up assessments were scheduled on days 14, 28, and 42. The patient showed notable improvement over 42 days, with affected BSA decreasing from 65% on day 1 to 12% on day 42 (Figure 2).

Key clinical indicators, including DLQI (from 20 to 3), SCORAD (from 55 to 18), EASI (from 27 to 5), SPS (from 3 to 0), sleep disturbance scale (from 7 to 1), and numerical itch scale (from 7 to 1), all demonstrated substantial reductions, indicating significant symptomatic relief (Table 2).

**Table 2:** The sequential change and improvement in signs of AD in terms of BSA and scoring systems for the second patient BSA: body surface area; DLQI: Dermatology Life Quality Index; SCORAD: Scoring Atopic Dermatitis; EASI: Eczema Area and Severity Index; SPS: Severity of Pruritus Scale; AD: atopic dermatitis

Follow-up	BSA involvement	DLQI	SCORAD	EASI	SPS	Sleep disturbance scale	Numerical itch scale
Day 1	65%	20	55	27	3	7	7
Day 14	65%	16	46	24	2	6	5
Day 28	37%	9	23	13	1	2	2
Day 42	12%	3	18	5	0	1	1

These cases underscore the potential of baricitinib in managing severe, cyclosporine-refractory AD.

## Discussion

AD is an immune-mediated inflammatory disorder affecting the Th2 pathway. IL-4, IL-5, and IL-13 are the major cytokines involved. JAKi, by selectively affecting the JAK-STAT pathways, downregulates these cytokines and is, hence, a novel therapeutic option for AD. Unlike autoimmune diseases like psoriasis or alopecia areata, where only one JAK pathway is dysregulated, AD involves increased signaling through all four JAKs (JAK1, JAK2, JAK3, and TYK2) [7].

The JAK/STAT pathway is a fast membrane-to-nucleus signaling pathway that regulates the expression of several important cytokines of inflammatory disorders and cancers [8]. Intracellular JAK proteins bind to type I/II cytokine receptors upon engagement by external ligands. This activation of the JAK proteins phosphorylates the STAT proteins, which dimerize and then translocate into the nucleus to regulate gene expression more precisely. Hence, JAKi is an effective treatment for rheumatic and dermatologic diseases. Unlike the disease-modifying antirheumatoid drugs targeting one or two specific cytokines, JAKi targets the cytokines in multiple immune pathways. Therefore, they possess rapid effects in both topical and oral forms [9].

The efficacy and safety of baricitinib monotherapy were assessed in a North American phase 3 clinical trial (BREEZE-AD5/NCT03435081) involving adults with moderate-to-severe AD who had demonstrated an inadequate response or intolerance to topical therapies. A total of 440 patients were randomly assigned in a 1:1:1 ratio to receive either a once-daily placebo, baricitinib 1 mg, or baricitinib 2 mg. The primary outcome was defined as the proportion of patients achieving at least a 75% reduction in the EASI-75 by week 16. A key secondary outcome included the proportion of patients attaining a validated Investigator Global Assessment (vIGA-AD) score of 0 (clear) or 1 (almost clear) with a minimum 2-point improvement. By week 16, EASI-75 was achieved by 8%, 13%, and 30% of patients in the placebo, baricitinib 1 mg, and baricitinib 2 mg groups, respectively, with a statistically significant difference observed for the 2 mg dose compared to placebo (p < 0.001). Similarly, a vIGA-AD score of 0/1 was attained by 5%, 13%, and 24% of patients, respectively (p < 0.001 for 2 mg vs. placebo). The safety profile remained consistent with previous studies evaluating baricitinib in AD. The short duration of the trial may limit the generalizability of the findings to real-world clinical practice. Baricitinib demonstrated efficacy in patients with moderate-to-severe AD, with no new safety concerns identified over the 16-week study period [10].

The efficacy and safety of baricitinib (4 mg/day) with topical corticosteroids were evaluated in 36 patients (≥15 years) with moderate-to-severe AD from August 2021 to September 2022. By weeks 4 and 12, median EASI reductions were 69.19% and 69.98%, with EASI-75 achieved in 38.89% and 33.33% of patients, respectively. Regional response varied, with the lower limbs showing the greatest improvement. Baseline EASI of the head and neck negatively correlated with early response, while higher baseline EASI in the lower limbs predicted better outcomes at week 12. Baricitinib reduced inflammatory markers and was well tolerated, with mild treatment-emergent adverse events, including creatine phosphokinase elevation (11.1%), herpes labialis (5.6%), and furuncle (8.3%). Findings suggest baricitinib provides clinical benefits similar to those in trials, with regional variability in response to predicting treatment outcomes [11].

A post hoc analysis of three phase III, 16-week trials (BREEZE-AD1, BREEZE-AD2, BREEZE-AD7) evaluated the effects of baricitinib on individual EASI subscores, excoriation, edema/papulation, erythema, and lichenification, in adults with moderate-to-severe AD receiving monotherapy or combination therapy with topical corticosteroids. Baricitinib 4 mg induced rapid and sustained improvement across all clinical signs, with significant effects on excoriation, edema/papulation, and erythema by week 1 (p < 0.001) and lichenification by week 2 (p < 0.001). Excoriation exhibited the most pronounced and sustained response, suggesting early disruption of the itch-scratch cycle. Findings highlight the efficacy of selective JAK 1/2 inhibition in achieving rapid control of AD-related inflammation [12].

Our experience underscores the efficacy and favorable safety profile of baricitinib in managing severe AD. Approved by the European Union for COVID-19 (in combination with remdesivir) and by the FDA for alopecia areata, baricitinib presents a viable treatment option for cyclosporine-resistant AD in India, where alternatives such as abrocitinib and dupilumab remain limited. Its accessibility and effectiveness position it as a valuable therapeutic addition for severe AD cases in this setting.

## Conclusions

In conclusion, managing AD remains challenging, particularly in patients unresponsive to conventional therapies. Baricitinib, a JAKi, has shown promise as an alternative, with significant improvements in disease severity, pruritus, and sleep quality observed in two adult cases without adverse effects. These findings highlight its potential as a safe and effective option for moderate-to-severe AD. However, long-term safety monitoring remains crucial, and further research is needed to better define its role in AD treatment.
